# Persistence in gestural communication predicts sociality in wild chimpanzees

**DOI:** 10.1007/s10071-018-1219-6

**Published:** 2018-10-19

**Authors:** Anna Ilona Roberts, Sam George Bradley Roberts

**Affiliations:** 1 Department of Psychology, University of Chester, Parkgate Road, Chester, CH1 4BJ UK; 2 School of Natural Sciences and Psychology, Liverpool John Moores University, Byrom Street, Liverpool, L3 3AF UK

**Keywords:** Chimpanzees, Gestural communication, Proximity, Grooming, Cooperation, Joint activity, Social bonds, Social networks, Elaboration, Repetition, Response, Evolutionary trade-off

## Abstract

A key challenge for primates is coordinating behaviour with conspecifics in large, complex social groups. Gestures play a key role in this process and chimpanzees show considerable flexibility communicating through single gestures, sequences of gestures interspersed with periods of response waiting (persistence), and rapid sequences where gestures are made in quick succession, too rapid for the response waiting to have occurred. The previous studies examined behavioural reactions to single gestures and sequences, but whether this complexity is associated with more complex sociality at the level of the dyad partner and the group as a whole is not well understood. We used social network analysis to examine how the production of single gestures and sequences of gestures was related to the duration of time spent in proximity and individual differences in proximity in wild East African chimpanzees (*Pan troglodytes schweinfurthii*). Pairs of chimpanzees that spent a longer duration of time in proximity had higher rates of persistence sequences, but not a higher rate of single gestures or rapid sequences. The duration of time spent in proximity was also related to the rate of responding to gestures, and response to gesture by activity change. These results suggest that communicative persistence and the type of response to gestures may play an important role in regulating social interactions in primate societies.

**Electronic supplementary material:**

The online version of this article (10.1007/s10071-018-1219-6) contains supplementary material, which is available to authorized users.

## Introduction

Primate social life has frequently been described as particularly complex in its nature, and when compared with other vertebrates, primates have unusually large brains for their body size (Dunbar 1993, 1998). Primate sociality is based on bonded social relationships where individuals repeatedly interact with the same group members in many different contexts (Freeberg et al. 2012). It has been proposed that the sociality of primates is cognitively demanding, leading to the evolution of large brains in both primates and hominins (Dunbar and Shultz 2007a). In particular, there is a strong positive correlation between group size and brain size in primates, and particularly neocortex size in relation to the rest of the brain (Dunbar 1993). Thus, primates living in larger groups have larger neorcortex ratios (Dunbar and Shultz 2007a). The relationship between brain size and group size may be influenced by the demands arising from maintaining social relationships in primates. Primates use grooming behaviour to maintain stable, long lasting, and differentiated social relationships with both related and unrelated individuals (Dunbar 2010). The time and cognitive demands arising from maintaining social relationships through grooming result in a multilevel group structure, with hierarchically nested layers of social bonds, delineated by decreasing amounts of time spent in grooming behaviour and proximity (Hill et al. 2008).

In addition, gestural communication, defined as voluntary movements of the arms, head, body postures, and locomotory gaits (Bard 1992; Hewes 1973; Roberts and Roberts 2017, 2018; Roberts et al. 2014a; Tomasello et al. 1994) is important in maintaining social relationships of primates (Bard 1992; Bard et al. 2014; Forrester 2008; Fröhlich et al. 2016; Genty et al. 2009; Gillespie-Lynch et al. 2013; Halina et al. 2013; Hewes 1973; Hobaiter and Byrne 2011a; Leavens et al. 2005; Liebal et al. 2004; Maestripieri 2005; McCarthy et al. 2013; Pika et al. 2005; Pollick and de Waal 2007; Roberts et al. 2012b, 2014a; Schneider et al. 2012; Scott 2013; Taglialatela et al. 2015; Tomasello et al. 1985, 1994). Gestural communication is particularly relevant for studies of social cognition, because gestures can flexibly influence social bonding and this may have important implications for the complexity of cognitive skills involved in managing of social relationship. In gestural communication, signallers have a goal and influence the recipient flexibly based on an understanding that recipients have goal states different from their own and these states can affect their behaviour (Tomasello and Zuberbühler 2002). In addition, gestures can coordinate social bonding behaviour by fulfilling social bonding function in itself by releasing social bonding neurohormones in the recipients (Dunbar 2010). For instance, greeting gestures when encountering each other after a period of separation can influence social bonding with the recipient and hence influence the duration of time spent in close proximity. Thus, gestural communication has an adaptive function and can coordinate social behaviour through influencing emotional states of the recipients (Spoor and Kelly 2004).

In particular, primate gestures that occur singly or in sequences can reveal the link between gestural communication and social bonding (Cartmill and Byrne 2007a; Genty and Byrne 2009; Hobaiter and Byrne 2011b; Leavens et al. 2005; Liebal et al. 2004; McCarthy et al. 2013; Roberts et al. 2012a, 2013, 2014a, b; Tanner 2004; Tanner and Perlman 2017; Tempelmann and Liebal 2012; Tomasello et al. 1994). Series of gestures made in anticipation of a response, as shown by persistence (Gómez 1996; Moore 2016; Scott-Phillips 2015a, b) may be important in social bonding in primates, because they influence behaviour directly (Cartmill and Byrne 2007a; Leavens et al. 2005; Roberts et al. 2013, 2014b). In sequences of gestural communication that are characterized by persistence, the signaller makes a gesture, pauses for 1–5 s to wait for a response, and then, if the response is not forthcoming, the signaller makes another gesture (Hobaiter and Byrne 2011b). Moreover, great apes can also make a ‘rapid sequence’ whereby several gestures are made in quick succession, too rapid for the response waiting to have taken place (Hobaiter and Byrne 2011b).

In gestural communication, the signaler modifies the production of the signals flexibly (Bates et al. 1979; Leavens et al. 2005; Tomasello et al. 1994). Observational and experimental studies in experimental tasks, and in conspecific social interactions, have showed that signalers can adjust their gestural communication in relation to the changes in the behaviour of the recipient (Cartmill and Byrne 2007a; Genty and Byrne 2009; Hobaiter and Byrne 2011b; Leavens et al. 2005; Liebal et al. 2004; McCarthy et al. 2013; Roberts et al. 2012a, 2013, 2014a, b; Tanner 2004; Tanner and Perlman 2017; Tempelmann and Liebal 2012; Tomasello et al. 1994). In experimental studies that manipulated the response consequences of ‘unsuccessful’ communication against a baseline of ‘successful’ communication, it was clearly demonstrated that apes can respond to the different behavioural states of the experimenter (Cartmill and Byrne 2007b; Leavens et al. 2005). For instance, individuals discontinued communicative attempts when the desired response was obtained and continued communicating when faced with an absence of a response (Cartmill and Byrne 2007a, 2010; Leavens et al. 2005; Roberts et al. 2012a, 2013, 2014b). Moreover, in a food finding task that required language-trained chimpanzees to guide a naïve human experimenter to a hidden food item, the chimpanzees coordinated their behaviour with the experimenter in a flexible way, based on the experimenter’s responses to the chimpanzees’ communication. The chimpanzees used nonindicative gestures such as bobbing when the experimenter accurately pointed to the food location and indicative gestures such as pointing when the experimenter pointed to a location where the food was not hidden (Roberts et al. 2014b). However, whilst the role of persistence in influencing the recipient’s behaviour has been shown in the previous studies, the role of persistence in social bonding is currently unclear. In addition, very little is known about the role of single gestures and rapid sequences in social bonding. Thus, the issue of whether great apes can use gestural communication flexibly to coordinate social behaviour with different types of social partners, and how this use relates to individual variation in sociality, remains unresolved.

Chimpanzees are an ideal species to examine the relationship between sociality and the production of single gestures, persistence, and rapid sequences in primates. Chimpanzees live in complex fission–fusion groups, where association dynamics are fluid and chimpanzees form temporary subgroups (‘parties’) that vary in size, composition, and duration (Goodall 1986). Due to this fission–fusion structure, patterns of interaction between pairs of chimpanzees can vary on daily basis. In this study, we examine the relationship between social interactions and the production of single gestures, persistence, and rapid sequences in wild East African chimpanzees (*Pan troglodytes schweinfurthii*) in Budongo Forest, Uganda, using Social Network Analysis (SNA). We examine how different types of communication (single gestures, rapid, and persistence sequences) are related to sociality. In this study, consistent with the previous research in this area (Lehmann et al. 2016; Sapolsky et al. 1997; Silk et al. 2010, 2013), we used proximity to measure differences in sociality between pairs of chimpanzees. We examined how these differences in sociality relate to patterns of communication between pairs of chimpanzees.

Through rapid sequences of gestures, signaler can induce compatible affect with the recipient and through synchronized affect, the rapid sequence can facilitate attentional and behavioural convergence between the dyad partners (Owren and Rendall 2001). In contrast, persistence sequence and single gesture can influence behaviour of the recipient by influencing their movement and attention to achieve a goal directly. It could be argued that single gestures and persistence sequence have evolved as a means to enable social bonding with dyad partners as they can influence behaviour of the recipient more flexibly than rapid sequence and this may have been accompanied by increase in brain size during the course of hominin evolution.

In this study, we explored the associations between proximity and these different types of gestural communication. Overall, we predict that that the duration of proximity between pairs of chimpanzees will be differentially associated with the rates of different types of gestural communication between these pairs of chimpanzees—single gestures, rapid sequences, and persistent sequences. Specifically, we predict that that single gesture and/or persistence sequences will be associated with a longer duration of time spent in proximity, whereas rapid sequence will be associated with a shorter duration of time spent in proximity (Hypothesis 1).

However, it is unclear whether single gestures, rapid, and persistence sequences differ in response types made to the gestures and this would indicate the nature of the influence of these communication types on the recipient. Thus, one aim of this study is to distinguish between types of gestural communication by examining type of gesture used in relation to response type to the gesture. Recipients can respond in a goal directed way by adjusting their behaviour to the goal conveyed in the gesture, but can also respond communicatively. Thus, we hypothesize that goal directed and communicative responses will be differentially associated with the type of communication (Hypothesis 2). Specifically, we predict that single gestures and/or persistence will be associated with goal directed response (by activity change), whereas rapid sequences will be associated with response by communication (visual, tactile gesture or vocalisation).

Furthermore, it is currently unclear whether the response to the gesture may be associated with the degree of sociality. Presence and type of response (e.g. goal directed or communicative) can indicate the willingness of the recipient to coordinate behaviour with the signaller and thus reflect the level of social bonding (Schneider et al. 2017; Wilke et al. 2017). Following on from Hypothesis 1, we hypothesize that the presence and type of response will be associated with sociality. Specifically, we predict that if responsiveness facilitates proximity, then we would expect a longer duration of time spent in proximity to be associated with higher rate of response present and a lower rate of response absence (Hypothesis 3). We also predict that a longer duration of time spent in proximity would be associated with a higher rate of response by activity change and a lower rate of response by communication (Hypothesis 4).

Finally, individuals have different positions in the social network, with central individuals spending a greater duration of time in proximity to other chimpanzees, as compared to less central individuals. Specifically, centrality in this study is based on the total duration of time that an individual focal chimpanzee spends in proximity to the other 11 focal chimpanzees, so captures information both about the number of social bonds a focal chimpanzee has, and time spent in proximity to these bonds. Social network analysis allows for the examination of factors associated with variation in dyadic relationships (Hypotheses 1–4) and also factors associated with individual differences in network centrality (Borgatti et al. 2013). Individual differences in sociality are associated with fitness outcomes in a range of primate species (Lehmann et al. 2016; Silk 2007; Silk et al. 2010). However, less is known about how these individual differences in sociality may relate to individual differences in communication patterns—both in terms of communication produced by the focal individuals, and communication received by the focal individuals. The previous research in this population of chimpanzees has suggested that more central individuals have different overall patterns of vocal and gestural communication to less central individuals (Roberts and Roberts 2016b), but this study did not examine whether individual differences in rates of single gestures or sequences are associated with centrality. As there is little prior research in this area, we do not make specific predictions for how the different types of gestures will be associated with centrality, but, instead, predict that, overall, the centrality of individual chimpanzees will be associated with the rate of singe, rapid and persistent gestural communication which they produce and they receive (Hypothesis 5).

The relationship between communication and social behaviour could arise simply as a relation between a behaviour that requires proximity with a metric of proximity. To avoid this possibility, in all analyses, we control for the duration of time spent in close proximity (all communication indices are calculated per duration of time spent within 10 m). Furthermore, in addition to the sequence type, biological factors such as reproductive status, age similarity, sex similarity, and kinship have been shown to influence patterns of social bonding between pairs of chimpanzees (Langergraber et al. 2009; Mitani 2009; Roberts and Roberts 2016b). Thus, we control for these biological factors in all the models.

## Methods

### Study site and subjects

The behaviour of East African chimpanzees (*Pan troglodytes schweinfurthii*) of the Sonso community at the Budongo Conservation Field Station, Budongo Forest Reserve in Uganda (latitude 1°37′–2°00′N; longitude: 31°22′–31°46′E) was observed in relation to communication and social relationships between March and June 2008, following subjects between 07:00 and 16:00 at least 5 days a week. The distance to the focal chimpanzee and the limb injuries of the chimpanzee can influence the frequency and type of gestural communication. Thus, from the community of approximately 74 individuals including 21 adult females and 10 adult males, a sample group of 12 adult focal subjects (6 adult males and 6 adult females) was chosen to ensure the lack of any limb injuries and in accordance with the level of habituation, simultaneously ensuring that age and rank classes were equally represented in the sample—see Table 1 (Roberts and Roberts 2016b) for demographic and sampling details of the focal chimpanzees. The study was noninvasive and the study methods were approved by the University of Stirling Ethics Committee. Full details of the study site, subjects, data collection, video analysis, and classification of gestures have been described previously (Roberts et al. 2014a), so only the key information is provided here.

**Table 1:** Focal ID, sex, year of birth, and reproductive status of the 12 focal subjects included in the study

Focal subject ID	Sex	Age	Female reproductive status	Total observation duration (minutes)
BB	Male	21	–	516
HW	Male	15	–	1030
KT	Male	15	–	1026
KU	Female	29	Pregnant	910
KW	Female	27	Nursing	510
ML	Female	33	Cycling	1118
MS	Male	17	–	524
NB^a^	Female	46	Cycling	500
NK^b^	Male	26	–	582
RH	Female	43	Nursing	1038
SQ	Male	17	–	554
ZM	Female	40	Cycling	710

Dominance based on unidirectional pant-grunt calls—for full details, see Roberts and Roberts (2016b)

^a^Alpha female

^b^Alpha male

### Data collection protocol

During 18-min focal follows consisting of nine scans (nine 2-min intervals), two types of social information were recorded. First, the association and activity patterns were recorded. These included the identity of individuals present within 10 m and more than 10 m away from the focal individual, and the identity, visual attention, distance, and activity of the nearest neighbour to the focal individual. Second, gestural communication to accompany the 18-min instantaneous sampling of association and behaviour patterns in the chimpanzees was recorded continuously using a digital video camera recorder.

Visual attention between the focal individual and the nearest neighbour was recorded using categories presented in Supplementary Information 2. We tested the similarity in association patterns between the scans taken at 2-min intervals, to examine the extent to which association patterns changed during the 18-min focal follows, and between one focal follow and the next. For full details of this analysis, see Roberts and Roberts (2016a, b). Briefly, the results demonstrated that the adjacent scans taken at 2 and 4 min of the 18-min sampling period yielded similar findings, and thus adjacent 2 min scans within a focal follow were treated as continuous data. However, the first scan (2 min) and final scan (18 min) during the focal follow differed both for 10-m associations and party-level associations. Thus, the association patterns change significantly over the course of an 18-min focal follow, meaning that each 18-min focal follow can be considered an independent sample of association patterns.

### Behavioural measures

First, we used the genetic relationships identified in the previous studies to classify pairs (dyads) of chimpanzees as kin or nonkin (Reynolds 2005), taking into account maternal kin relations only (relatedness 0.5). We classified dyads of chimpanzees as belonging to the same (5 years or less age difference) or a different (above 5 years age difference) age class (Mitani et al. 2002) and also according to reproductive and sex similarity. The details of the categorization of attribute data are provided in Table 2.

**Table 2:** Variables included in the models

Independent variable	Definition	Frequencies or mean ± SD/95% CI (duration/frequency per hour spent within 10 m)
Persistence sequence	A series of gestures whereby there are pauses of 1–5 s between consecutive gestures	0.11 ± 0.45, [0.03, 0.18]
Single gesture	A single gesture that is not made in series and where there is at least 30 s to the next consecutive gesture	1.27 ± 4.07, [0.57, 1.97]
Rapid sequence	A series of gestures without pauses between consecutive gestures	0.45 ± 1.30, [0.23, 0.68]
Sex difference	Sex difference between focal subject and the recipient (0 = different sex: male–female or female–male, 1 = same sex: male-male or female–female)	0 = 60, 1 = 60
Age difference	Age difference between focal subject and the recipient (0 = different age: more than 5 years age difference between individuals in the dyad, 1 = same age: no more than 5 years age difference between individuals in the dyad)	0 = 102, 1 = 30
Oestrous similarity	Reproductive state difference between focal subject and the recipient (0 = reproductively inactive: unoestrous female–unoestrous female, unoestrous female-oestrous female, oestrous female-oestrus female, unoestrous female–male, male–male; 1 = reproductively active: male–oestrous female)	0 = 96, 1 = 36
Maternal kinship	Maternal kinship presence between focal subject and the recipient (0 = unrelated dyad, 1 = mother–son; son–mother)	0 = 126, 1 = 6
Proximity	Duration of time individual spent in proximity within 10 m, per hour spent in the same party	23.26 ± 1.22, [20.84, 25.69]
Response by activity change	Change of behaviour by means of goal directed response, whereby recipient performs some action that conforms to the goal of the signaller (e.g. starts to groom)	0.58 ± 1.80, [0.26, 0.89]
Response by vocalisation	Change of behaviour by means of vocalisation (production of sound via vocal tract) by the recipient, which is not followed by goal directed action towards signaller (e.g. pant-grunt during travel, whereby signallers travel before and after the pant-grunt)	0.47 ± 2.02, [0.12, 0.82]
Response by visual or tactile gesture	Change of behaviour by means of visual or tactile gesture which excludes production of sound by the recipient via vocal tract. This behaviour is not followed by goal directed action towards signaller (e.g., embrace during travel, whereby signallers travel before and after the embrace)	0.08 ± 0.40, [0.01, 0.14]

Second, to establish the rates of gestures between dyads, the video footage was viewed on a television and the cases of nonverbal behaviour that were identified were coded as an act of gestural communication if they met following criteria: (1) the nonverbal behaviour was an expressive movement of the limbs or head and body posture that was mechanically ineffective, (2) the behaviour was communicative by nonmechanical means (i.e., consistently produced a change in the behaviour of recipient or facilitated maintenance of activity, e.g., grooming). Whilst the criterion of ‘nonmechanical means’ did not exclude cases of physical bodily movement by the signaller of a social partner, it was important that such cases had a communicative purpose, i.e., rather than just move the body part of the social partner physically, these cases also displayed communicative purpose, For example during grooming, the light touch of the body and subsequent slight displacement of the body part also meant the desire for the social partner to move the body part.

Next, behaviour had to be goal directed to be considered intentional (Bard 1992; Bates et al. 1979). The intentionality of gestures was coded sensu Tomasello et al. (1985) who gave the following example to explain intentionality of gestures: ‘a child might be struggling to open a cabinet, crying and whining as s/he struggles. Seeing this, the mother might come to the rescue and open the cabinet. This is a perlocutionary act, because, while communication may be said to have occurred, the “sender” (the child) did not intentionally direct any behaviour towards the mother. If, on the other hand, the child has turned its attention from the cabinet to the mother and whined at her, the whining now becomes a social-communicatory act with the intention of obtaining adult aid’. Operationally, thus, one clear evidence for intentionality of gestures comes from the presence of an audience and visual attention between signaller and the recipient during production of the gesture. In this data set, all cases of gesturing included the presence of an audience in close proximity (Supplementary Information 1 and 2), so the intentionality of the gestures in this data set was not differentiated by the presence of the audience. In addition, the presence and absence of bodily orientation before and during the gesture were coded to establish intentionality of gestures (see Supplementary Information 2 for details for each gesture type). The presence and absence of communicative persistence was also coded in this paper following communicative persistence sensu Hobaiter and Byrne (2011a) and Townsend et al. (2016). To establish communicative persistence, gesture events were scored in accordance to whether they occurred singly or in sequences, defined as one or more than one gesture made consecutively by one individual, towards the same recipient, with the same goal, within the same context, and made within a maximum of 30 s interval to ensure independence. Following the classification by Hobaiter and Byrne (2011b), persistence of gesturing is when the chimpanzee produces one gesture or a gesture sequence, then, after a period of response waiting (1–5 s), they produce another gesture—here, such instances are termed a ‘persistence sequence’. However, when a chimpanzee produces a sequence and there is no intermittent pause between gestures, then the chimpanzee has not persisted—here, such instances are here termed a ‘rapid sequence’. Supplementary Information 2 contains detailed information for the percentages of each gesture type occurring within each sequence type. Moreover, Supplementary Information 1 (Table 2) provides the number of cases of single gestures, persistence, and rapid sequences per each focal subject separately. The panthoot behaviour is broadcast at a wider audience, and within social network analysis, we counted all individuals present within 10 m as recipients of any gestures accompanied by pant hoots produced by the focal subject. The identity of the recipients of the panthoot was taken from the scan sample recorded every 2 min.

A random sample of 50 sequences of gestures was coded by a second coder for intentionality (response waiting and persistence) and the Cohen’s Kappa coefficient showed good reliability (*K* = 0.74) (Bakeman and Gottman 1997). In this sample of reliability coding of persistence, one requirement for categorizing the event as persistence was the presence of mutual bodily orientation between the signaller and the recipient. Thus, in this sample, response waiting and persistence co-occurred in all the cases of gesturing. Furthermore, a random sample of 55 gestures was coded by a second coder for response type (response presence or absence, response by activity change, response by vocalisation, and response by visual or tactile gesture). Cohen’s Kappa coefficients were calculated for each response type separately, based on whether the coder judged each category of response to be present or absent in a gesture sequence (e.g., was response by vocalisation present in a specific gesture sequence). Reliability was excellent for all the response types: response presence or absence (*K* = 0.93), response by activity change (*K* = 0.89), response by vocalisation (*K* = 0.84), and response by visual or tactile gesture (*K* = 0.85).

Having established the independence of the data collection protocol, the behavioural measures for each dyad of the signaller and the recipient were calculated in the following manner:

### The dyadic communication measure

The dyadic communication measure (CA) is the rate at which focal subject A communicated to nonfocal subject B when B was in close proximity (within 10 m) to focal subject A, per hour spent within 10 m of the nonfocal subject B, or:

$${\text{C}}{{\text{A}}_{{\text{AB}}}}={\text{(}}{C_{{\text{AB}}}} \times {\text{ }}60{\text{)}}/P{10_{{\text{AB}}}} \times 2,$$

where *C*_AB_ = the number of times A communicated with B when in close proximity (within 10 m) to B, P10_AB_ = the number of times A was in close proximity (within 10 m) to B, 2 = duration of instantaneous subsample interval in minutes, and 60 = the number of minutes in an hour.

The CA was calculated separately for single gestures, rapid sequences, and persistence sequences, giving a rate of gesturing for each of these three types of gestures, per hour a dyad pair spent in close proximity.

### Social network analysis (SNA)

The behavioural measures were entered into a network matrix consisting of 12 rows and 12 columns, with each row and column designating a different focal chimpanzee. In this analysis, only data on 132 focal and nonfocal subject dyads were included in the analysis, excluding any data where the recipient was not a focal subject in this study. The number of entries or mean ± SD for each behavioural measure are provided in Table 2. The values in each cell of the matrix represented the value for communication or proximity for a specific pair of chimpanzees (e.g., the rate of persistence sequence between Bwoba and Hawa, per hour spent within 10 m). These networks were weighted—i.e., each cell consisted of a continuous value representing that behaviour, rather than a 1 or a 0 indicating the presence or absence of a tie. Furthermore, the networks were directed in that the rate of gestures by Bwoba that were directed to Hawa may be different from the rate of gestures by Hawa that were directed to Bwoba.

The observations that make up network data are not independent of each other, and thus, in general, standard inferential statistics cannot be used on network data. Instead, a set of analyses using randomisation (or permutation) tests have been developed where the observed value is compared against a distribution of values generated by a large number of random permutations of the data. The proportion of random permutations in which a value as large (or as small) as the one observed is then calculated, and this provides the *p* value of the test (Borgatti et al. 2013). We used multiple regression quadratic assignment procedure (MRQAP) to examine the relationships between the networks (Borgatti et al. 2013). MRQAP regression is similar to standard regression in that it allows for the examination of the effect of a number of independent variables (e.g., gestural communication networks) on an outcome variable (e.g., proximity network). Several different types of MRQAP regression are available and we used Double Dekker semi-partialling MRQAP regression, which is more robust against the effects of network autocorrelation and skewness in the data (Dekker et al. 2007). The number of permutations used in this analysis was 2000. All data transformations and analyses were carried out using UCINET 6 for Windows (Borgatti et al. 2014).

## Results

### Intentionality of gestural communication

We examined a total of 545 sequences (1044 instances of gestures) performed by 12 focal adult individuals towards other focal and nonfocal adult individuals to examine the extent to which the gestures presented in this data set were intentional. The percentage of association between each gesture type separately and indices of intentionality is given in Supplementary Information 1, Table 1. Moreover, frequencies of gesture events within these categories are provided in Supplementary Information 2. In this sample (consisting of adult to adult gestures only), the mean percentage ± SD [95% CI] of cases of all gesture types associated with the presence of bodily orientation by the signaller towards the recipient during the production of the gesture was 91.5 ± 18.5%, [87, 95]. The mean percentage ± SD [95% CI] of cases of all gesture types associated with the presence of recipients’ bodily orientation towards signaller, when the signaller’s bodily orientation towards the recipient was absent, was 6.9 ± 15.4% [3, 10]. Finally, the mean percentage ± SD [95% CI] of cases of all gesture types where neither signaller nor the recipient was bodily oriented towards one another during production of the gesture was 1.5 ± 11% [0, 3]. Using visual attention as a criterion for intentionality, these results show that the gestures in our data set were intentional (Bard 1992; Bates et al. 1979).

In this paper, sequences were categorized as either single gestures, persistence sequences or rapid sequences following Hobaiter and Byrne (2011b), taking into account both manual and bodily gestures (Roberts et al. 2012b, 2014a). Per focal individual, the mean number ± SD [95% CI] of single gestures was 32.0 ± 32, [11.69, 52.47], for persistence sequences was 4.41 ± 5.85, [0.69, 8.13] and for rapid sequences was 8.9 ± 9.09, [3.14, 14.69]—see also Supplementary Information 1, Table 2 for frequency of single gestures, persistence, and rapid sequences for each focal subject separately.

In this study, we used two main sets of analyses: multiple regression quadratic assignment procedures (MRQAP), and node-level regression. The description of all the variables included in these models are provided in Table 2. In all analyses, the age, sex, reproductive status, and kinship were included in the models, including the recipient of the gesture entered as a dyad partner in all the models. Full details of the models including all variables are provided in Tables 3, 4, 5, 6, 7, 8, and 9.

**Table 3:** MRQAP regression models showing predictors of proximity (duration of time spent within 10 m, per hour spent in the same party) by sequence type of gestures between *N* = 12, 132 dyadic relationships of the chimpanzees

Attribute category/rate of gesture sequence per hour spent in close proximity	Standardized coefficient	Standard error	*p*
Age similarity	0.162	3.658	0.060
Sex similarity	− 0.091	3.760	0.239
Kinship similarity	0.065	6.742	0.258
Oestrous similarity	0.006	4.328	0.487
Rapid sequence	− 0.025	1.107	0.389
Single gesture	0.110	0.370	0.138
Persistence sequence	0.164	3.109	**0.034**

Significant *p* values are indicated in bold

**Table 4:** MRQAP regression models showing predictors of rapid sequence (rate of production per hour spent within 10 m) by rate of response to the gesture between *N* = 12, 132 dyadic relationships of the chimpanzees

Attribute category/rate of gesture sequence per hour spent in close proximity	Standardized coefficient	Standard error	*p*
Age similarity	0.010	0.160	0.386
Sex similarity	− 0.057	0.169	0.176
Kinship similarity	− 0.037	0.283	0.142
Oestrous similarity	− 0.060	0.193	0.171
Response by visual or tactile gesture	0.006	0.353	0.471
Response by activity change	− 0.067	0.084	0.271
Response by vocalisation	0.857	0.065	**0.001**

Significant *p* values are indicated in bold

**Table 5:** MRQAP regression models showing predictors of persistence sequence (rate of production per hour spent within 10 m) by rate of response to the gesture between *N* = 12, 132 dyadic relationships of the chimpanzees

Attribute category/rate of gesture sequence per hour spent in close proximity	Standardized coefficient	Standard error	*p*
Age similarity	− 0.029	0.086	0.373
Sex similarity	0.042	0.086	0.327
Kinship similarity	− 0.015	0.152	0.437
Oestrous similarity	0.053	0.095	0.275
Response by visual or tactile gesture	− 0.754	0.181	**0.001**
Response by activity change	1.132	0.048	**0.001**
Response by vocalisation	0.067	0.019	0.134

Significant *p* values are indicated in bold

**Table 6:** MRQAP regression models showing predictors of single gesture (rate of production per hour spent within 10 m) by rate of response to the gesture between *N* = 12, 132 dyadic relationships of the chimpanzees

Attribute category/rate of gesture sequence per hour spent in close proximity	Standardized coefficient	Standard error	*p*
Age similarity	0.103	0.492	**0.017**
Sex similarity	0.047	0.493	0.195
Kinship similarity	0.002	0.844	0.373
Oestrous similarity	0.037	0.534	0.282
Response by visual or tactile gesture	0.392	0.901	**0.001**
Response by activity change	0.488	0.247	**0.001**
Response by vocalisation	0.068	0.100	0.083

Significant *P* values are indicated in bold

**Table 7:** MRQAP regression models showing predictors of proximity (duration spent within 10 m per hour spent in the same party) by rate of response present or absent to the gesture between *N* = 12, 132 dyadic relationships of the chimpanzees

Attribute category/rate of gesture sequence per hour spent in close proximity	Standardized coefficient	Standard error	*p*
Age similarity	0.149	3.748	0.078
Sex similarity	− 0.059	3.704	0.321
Kinship similarity	0.064	6.619	0.252
Oestrous similarity	0.030	4.282	0.397
Response absent	0.006	0.573	0.466
Response present	0.178	0.380	**0.026**

Significant *p* values are indicated in bold

**Table 8:** MRQAP regression models showing predictors of proximity (duration spent within 10 m per hour spent in the same party) by rate of response to the gesture between *N* = 12, 132 dyadic relationships of the chimpanzees

Attribute category/rate of gesture sequence per hour spent in close proximity	Standardized coefficient	Standard error	*p*
Age similarity	0.198	3.887	0.026
Sex similarity	− 0.127	3.802	0.154
Kinship similarity	0.063	6.539	0.239
Oestrous similarity	− 0.004	4.093	0.479
Response by visual or tactile gesture	− 0.391	6.567	**0.012**
Response by activity change	0.603	1.746	**0.002**
Response by vocalisation	− 0.088	0.761	0.198

Significant *p* values are indicated in bold

**Table 9:** Node-level regression models predicting proximity out degree (overall durations of time spent in proximity within 10 m, per hour dyad spent in the same party)

Attribute category/agreement in gesture repertoires	Standardized coefficient	*P*
Reproductive state of female	− 1.605	**0.025**
Kinship	0.359	0.250
Sex/ age	− 0.492	0.210
Rapid sequence outdegree	− 0.112	0.466
Rapid sequence indegree	− 0.046	0.471
Single gesture outdegree	0.255	0.431
Single gesture indegree	− 0.691	0.166
Persistence sequence outdegree	− 0.208	0.389
Persistence sequence indegree	1.858	**0.015**

Outdegree refers to behaviours directed by the focal chimpanzee to conspecifics, whilst indegree refers to behaviours directed by conspecifics towards the focal chimpanzee. Based on 12 chimpanzees, significant *p* values are indicated in bold

### Type of sequence and proximity (Hypothesis 1)

**Fig. 1:**
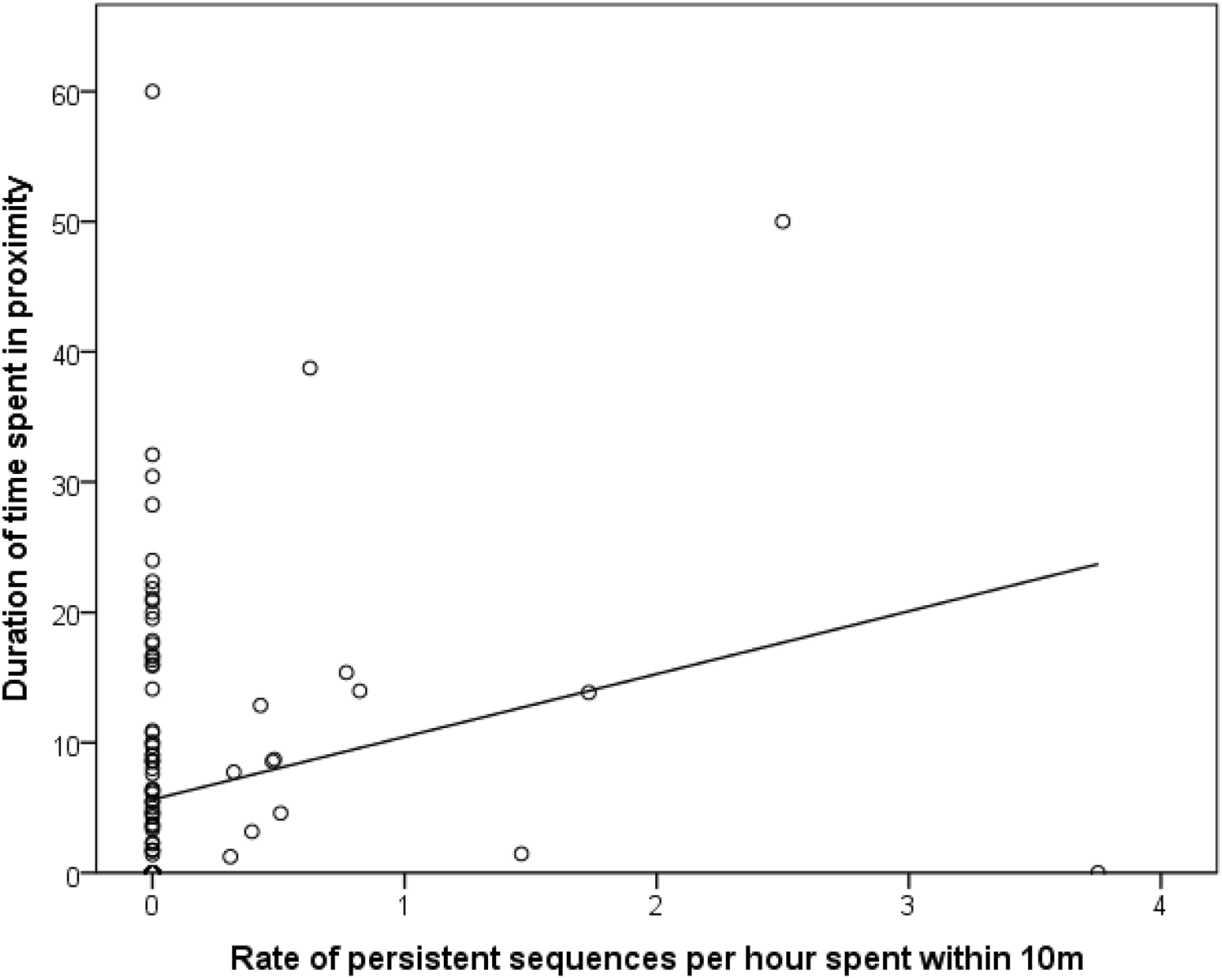
Duration of time spent in proximity (time in mins spent within 10 m, per hour spent in the same party) and rate of persistence sequences in dyads of chimpanzees (*n* = 132)

We used MRQAP to examine the relationship between duration of time spent in proximity (within 10 m per hour spent in the same party), the rate of production of gestures (frequency per hour spent within 10 m) and demography (Table 3). Proximity was positively associated with the rate of persistence sequence between dyads (*β* = 0.164, *p* = 0.034; Fig. 1). In contrast, the rate of rapid sequences (*β* = − 0.025, *p* = 0.389) or single gestures (*β* = 0.110, *p* = 0.138) was not associated with proximity.

### Type of sequence and type of response (Hypothesis 2)

We then examined how the rate of response type to the gestures (response by visual or tactile gesture, response by vocalisation, and response by activity change) was associated with the type of sequence (rapid sequence, persistence sequence, and single gesture) (Tables 4, 5, 6). There was a positive association between response by vocalisation and rapid sequence (*β* = 0.857, *p* = 0.001). Moreover, there was a positive association between response by activity change and persistence (*β* = 1.132, *p* = 0.001) but a negative association between response by tactile or visual gesture and persistence (*β* = − 0.754, *p* = 0.001). Finally, there was a positive association between single gesture and response type by activity change (*β* = 0.488, *p* = 0.001) and a positive association between single gesture and response by visual or tactile gesture (*β* = 0.392, *p* = 0.001).

### Presence and absence of response and proximity (Hypothesis 3)

We next examined how the rate of response type to the gestures (response presence and absence) was associated with the duration of time spent in proximity (Table 7) There was a significant positive association between proximity and response presence (*β* = 0.178, *p* = 0.026). However, there was no significant relationship between proximity and response absence (*β* = 0.006, *p* = 0.466).

### Type of response and proximity (Hypothesis 4)

In the next analysis, we examined how the rate of response type to the gestures (response by visual or tactile, gesture, response by vocalisation, and response by activity change) was associated with proximity (Table 8). There was a significant positive association between proximity and response by activity change (*β* = 0.603, *p* = 0.002). In addition, there was a significant negative association between proximity and response by visual or tactile gesture (*β* = − 0.391, *p* = 0.012).

### Sequence network size and centrality in proximity network (Hypothesis 5)

Finally, we used node-level regressions to examine the association between gesture sequences (rapid and persistence), single gestures, and individual position in the proximity network (centrality out degree). Outdegree refers to behaviours directed by the focal chimpanzee to conspecifics, whilst indegree refers to behaviours directed by conspecifics towards the focal chimpanzee. The network values can vary between dyad A to B and B to A (e.g., the rate of gestures directed from Bwoba to Hawa can be different from the rate of gestures directed from Hawa to Bwoba); therefore, indegree and outdegree are calculated separately. All analyses controlled for the duration of time spent in proximity to oestrus females, time spent in proximity to kin, and the age and sex of the focal chimpanzee. There was a positive association between centrality and persistence sequence in degree (*β* = 1.858, *p* = 0.015, Table 9). Thus, individual chimpanzees who spent a longer duration of time in proximity to others received a higher rate of persistence sequences directed at them.

## Discussion

An important aspect in understanding the evolution of complex sociality in humans is to understand the role of primate sequences of gestures in social bonding at the level of the dyad and the group. Chimpanzees produce single gestures (produced singly rather than in series), persistence sequences (series of gestures interspersed with periods of response waiting), and rapid sequences (series of gestures made in quick succession without periods of response waiting) (Hobaiter and Byrne 2011b). Recent theoretical accounts emphasize the role of gestures not purely as a means of information transfer (Seyfarth et al. 2010), but as a time-efficient mechanism of social bonding (Dunbar 2012). However, studies to date have examined the characteristics of gesture in isolation from the social system (Bard 2017; Bard et al. 2017; Byrne et al. 2017; Leavens et al. 2017). Thus, the mechanisms through which gesture sequences can be associated with more complex social systems remain unresolved. In this study, we used a sample of 12 wild chimpanzees to examine how the production of gesture sequences was related to patterns of sociality at both the individual and group levels. This extends the previous research which has focused on the association between the type of gesture sequence and the response of the recipient. Overall, the results demonstrate a significant association between patterns of proximity between pairs of chimpanzees and rates of communicative persistence.

We argued that persistence sequences and single gestures may be associated with influencing the recipient’s attention and behaviour, whereas rapid sequences may be associated with communicative responses. Thus, broadly, we predicted that single gestures and persistence sequences would show different patterns of associations to rapid sequences in relation to proximity (Hypothesis 1), response to the gesture (Hypothesis 2), and the network centrality of the signaller (Hypothesis 5). Overall, this set of hypotheses was not supported by the results.

Specifically, a longer duration of proximity, per hour spent within 10 m, was associated with a higher rate of persistence sequences, but not a higher rate of single gestures as predicted. Furthermore, there was not a significant association between proximity and the rate of rapid sequences (Hypothesis 1). Contrary to Hypothesis 2, a higher rate of response by communication was associated with both single gestures (response by visual or tactile gesture) and rapid sequences (response by vocalisation). Finally, individuals with higher centrality in the network (individual chimpanzees who spent a longer duration of time in proximity to others) did not produce rapid, single, or persistence sequences at a higher rate than less central individuals (Hypothesis 5). Overall, the results do not provide evidence for a clear distinction between single gestures and persistence sequences, versus rapid sequences, in relation to proximity at either a dyadic or network level. The result also do not support this distinction in relation to the response to the gesture, in terms of a goal directed response (a change in activity by the recipient) for single and persistence gestures and a communicative response that may be underpinned by an emotional or affective component for rapid sequences.

One reason why single gestures were not associated with proximity and response in the way predicted may because in this study, these gestures were not differentiated by the presence or absence of a response to the gesture. For instance, use of single gestures when a response is present may indicate stronger social bonds when considered separately from use of a single gesture when response was absent (Roberts et al. 2012a). Thus, by examining single gestures all together, the role of single gestures in managing social relationships may have been obscured. In addition, rapid sequences were not negatively associated with proximity as predicted. In our previous studies, we showed that loud auditory gestures accompanied by synchronized panthoot calls occurred between individuals who spent shorter durations of time in proximity (Roberts and Roberts 2016b). By not taking synchrony in communication during production of rapid sequences into account, these rapid sequences may not have as strong as effect on social bonding with individuals who have infrequent interactions with the focal chimpanzee. Further research is required to clarify how different types of gestures relate to sociality and how this relationship may be influenced by factors such as the response to the gesture, the age of the signaler, with less use of persistence sequences in older chimpanzees (Hobaiter and Byrne 2011b) and the behavioural context in which the gesture occurs (Hobaiter and Byrne 2011a; Roberts et al. 2013).

The two other hypotheses related to how the duration of proximity between pairs of chimpanzees is related to the presence and absence of a response to gestural communication (Hypothesis 3) and the type of response (Hypothesis 4), rather than rates of different types of gestural communication. Hypothesis 3 was partially supported, in that a longer duration of proximity was associated with a higher rate responding to the gesture (response present), but was not associated with a lower rate of response absence. Hypothesis 4 was supported—a longer duration of proximity was associated with a higher rate of response by activity change and a lower rate of response by communication (visual or tactile). Furthermore, the rate of persistence sequences was associated with a longer duration of proximity between dyads. These results suggest that one possible function of communication between individuals who spend a longer duration of time in proximity is to enable responsiveness by influencing the behaviour of the recipient. Thus, one important dimension of complex social interactions is the degree of successful inter-individual adjustment between interactants, enabling them to coordinate joint activities such as mutual grooming, travel, or mating through intentional gesturing (Froehlich et al. 2016; Roberts and Roberts 2015; Roberts et al. 2014a). Recent studies have provided evidence that gestural communication responded to by the recipient appears to be related to stronger social bonds than communication which has not been responded to (Schneider et al. 2017). Therefore, one reason why individuals who spent longer durations of time in proximity use persistence sequences may be, because they can influence the recipient flexibly to facilitate social interaction and achieve their communication goal (Roberts et al. 2014a).

In line with the previous research in this area (Lehmann et al. 2016; Sapolsky et al. 1997; Silk et al. 2010, 2013), we used proximity to measure the level of sociality of pairs of chimpanzees. This allowed for the association between one measure of sociality and rates of different types of gestural communication to be explored. However, different types of social behaviours may play different roles in social cohesion in primates. For instance, the role of grooming in primate social relationships is well established (Dunbar 2010), but the role of other joint behaviours such as joint travel or joint feeding is less clear (Gruber and Zuberbühler 2013; King et al. 2011). Similarly, in humans, cooperative contexts whereby actors co-regulate behaviour with one another to achieve common goal (e.g., joint travel) reflect stronger social bonding than other contexts (Pollet et al. 2013; Wolf et al. 2016). However, whether these different dimensions of sociality are differentially linked to gestural communication within dyads is unclear from this study and future work could examine specific instances of behaviour (e.g., grooming interactions, travel initiation) to explore the role of different types of gestural communication in coordinating this behaviour (Fedurek et al. 2015).

In addition to examining factors associated with variation in dyadic relationships, we also examined factors associated with individual differences in sociality. Consistent with the previous findings (Lehmann et al. 2016; Silk 2007; Silk et al. 2010), individual chimpanzees differed in the amount of time which they spent in proximity to the 11 other focal chimpanzees—measured in this study as network centrality. As discussed above, overall there were no significant associations between centrality and the rates of single, rapid, and persistence sequences produced by the focal individuals and thus Hypothesis 5 was rejected. The one significant finding was that more central individuals received persistence sequences at a higher rate, but not rapid sequences or single gestures at a higher rate. One interpretation of this finding could be that central individuals have greater demands on their social time and attention as they spend a longer duration of time in proximity to others. Thus, when communicating with these central individuals, signallers may use persistence sequences at a higher rate to increase the probability that the goal of their communication is met (Hobaiter and Byrne 2011b). However, this result should be treated with caution as only one of the six relationships examined (single gestures, rapid, and persistence sequences produced and received) was significant, suggesting that centrality might not play a key role in linking gesture use and sociality. Further research is necessary to examine whether individual differences in sociality are reliability associated with individual differences in patterns of vocal and gestural communication (Roberts and Roberts 2016b).

The conclusions drawn in this study could be influenced by the uneven representation of different gestures within data set. The previous studies which employed the continuous observation of gestures have ranged between 3 (Hobaiter et al. 2017) and 5 h (Wilke et al. 2017) of observation of each focal individual during study period. In the current study, we observed 12 focal subjects from a single study group for a mean duration of 12.52 h per each focal chimpanzee, ranging between 8.3 and 18.6 h (considering the video data collected in parallel with the socio-ecological samples during the last data collection season). However, the sampling of focal individuals was uneven and single gestures and sequences vary in their occurrence rates. For instance, in this study, there were 160 sequences of different types, whereas there were 385 single gestures. Similarly, gesture types were not distributed evenly across categories, as a majority of gesture types were confined to most common occurrence categories. Thus, whilst the results are broadly in line linking gestural communication with sociality and coordination of behaviour in primates (Byrne et al. 2017; Leavens et al. 2005; Roberts et al. 2014b), further research is needed to explore how gestural communication is associated with sociality in other chimpanzee communities and other primate species. This further research could focus on compiling a data set whereby gesture sequences and gesture types would be represented more equally. Furthermore, whilst we explored associations between sociality and gestural communication, we could not demonstrate a causal relationship between gestural communication and a longer duration of proximity between pairs of chimpanzees. Research examining how specific types of gestural communication are associated with the durations of specific instances of social behaviour would be needed to establish such a causal relationship. Many gestures are produced in the context of grooming (Byrne et al. 2017; Roberts et al. 2012a) and one promising area for future research would be to examine whether specific types of gestures given in grooming contexts are associated with longer grooming bouts or reduced probability of defecting to an alternative grooming partner (Fedurek et al. 2015; Kaburu and Newton-Fisher 2016).

The predictability of conspecifics’ behaviour is a major modulator of stress in group living animals (Seyfarth and Cheney 2013) and greater use of communicative persistence may reduce this stress by increasing the likelihood of the recipient responding appropriately to the gesture. This is especially important as gestural communication can be used in both affiliative and agonistic contexts in close proximity (Roberts et al. 2012b), and thus, communicative persistence may lead to greater coordination of behaviour between pairs of chimpanzees. The previous research has focused on how intentionality in gestural communication is related to the recipients’ response and comprehension of signaling, both in relation to human and conspecific recipients (Cartmill and Byrne 2007a; Leavens et al. 2005; Roberts et al. 2013, 2014b). Whilst this research has detailed the extent to which chimpanzees can flexibility adjust their communication, and explored how sensitive these adjustments are to different aspects of the recipients response, it has not demonstrated how this flexibility in communication helps chimpanzees meet the key adaptive challenges faced by group living animals—maintaining a differentiated set of stable, long-term social relationships, and responding appropriately to others (Dunbar and Shultz 2007a). If the key driving force of brain evolution in both primates and hominins has been the evolution of complex social relationships rather than ecological factors (Dunbar and Shultz 2007b), the cognitive skills underpinning flexibility in communication should enable primates to meet these social challenges. The current results suggest that communicative persistence may enable greater levels of behavioural coordination when interacting at close proximity and thus longer durations of proximity and affiliative activities such as grooming.

To conclude, the ability to successfully coordinate social behaviour through gestural signals with conspecifics is a key aspect of successful group living (Seyfarth and Cheney 2013). The findings of this study demonstrate that persistence sequences in gestural communication are associated with sociality, as measured by a longer duration of proximity, and may help chimpanzees meet the challenges of group living. Individual variation in the strength of social bonds in primates is strongly linked to fitness outcomes (Silk 2007) and our results suggest that persistence in gestural communication may play an important role in explaining some of this individual variation in social relationships.

## Electronic supplementary material

Below is the link to the electronic supplementary material.

Supplementary material 1 (DOCX 24 KB)

Supplementary material 2 (DOCX 17 KB)
