# Supplementary material for: Persistence in gestural communication predicts sociality in wild chimpanzees
Source: Anim Cogn. 2018 Oct 19;22(5):605–18. doi: 10.1007/s10071-018-1219-6 (PMC6689904; doi:10.1007/s10071-018-1219-6)
Supplement: Supplementary file 1 — Supplementary material 1 (DOCX 24 KB) [file 10071_2018_1219_MOESM1_ESM.docx]

**Persistence in gestural communication predicts complex sociality in wild chimpanzee**

**Supplementary Information 1**

**Animal Cognition**

Anna Ilona Roberts, Sam George Bradley Roberts

Supplementary Table 1. Intentionality of gestures by gesture type (expressed as percentage of total events)

| Gesture type | Audience presence | Sequence type | | | Bodily orientation during production of the gesture | | |
| --- | --- | --- | --- | --- | --- | --- | --- |
|  |  | Rapid | Persistence | Single gesture | Signaller and recipient out of each other’s view | Recipient oriented towards signaller when signaller not attending | Signaller oriented towards the recipient |
| Arm beckon | 100% | 0% | 100% | 0% | 0% | 0% | 100% |
| Arm flap | 100% | 50% | 0% | 50% | 0% | 20% | 80% |
| Arm raise | 100% | 0% | 0% | 100% | 0% | 0% | 100% |
| Beat | 100% | 100% | 0% | 0% | 0% | 0% | 100% |
| Bite | 100% | 100% | 0% | 0% | 0% | 0% | 100% |
| Bob | 100% | 36% | 36% | 27% | 0% | 0% | 100% |
| Bounce | 100% | 19% | 38% | 44% | 0% | 8% | 92% |
| Bow | 100% | 46% | 31% | 23% | 0% | 10% | 90% |
| Break | 100% | 100% | 0% | 0% | 0% | 0% | 100% |
| Clip by hand | 100% | 17% | 33% | 50% | 0% | 0% | 100% |
| Clip by mouth | 100% | 0% | 50% | 50% | 0% | 0% | 100% |
| Crouch | 100% | 33% | 50% | 17% | 0% | 0% | 100% |
| Crouch run | 100% | 58% | 13% | 29% | 0% | 11% | 89% |
| Crouch walk | 100% | 49% | 21% | 31% | 0% | 14% | 86% |
| Dangle | 100% | 75% | 13% | 13% | 17% | 17% | 67% |
| Drag object | 100% | 100% | 0% | 0% |  |  |  |
| Drag self | 100% | 0% | 100% | 0% | 0% | 0% | 100% |
| Drum | 100% | 89% | 5% | 5% | 0% | 20% | 80% |
| Embrace | 100% | 77% | 0% | 23% | 0% | 23% | 77% |
| Forceful extend | 100% | 0% | 50% | 50% | 0% | 0% | 100% |
| Grab | 100% | 50% | 0% | 50% | 0% | 0% | 100% |
| Hand bend | 100% | 25% | 38% | 38% | 0% | 0% | 100% |
| Hit object | 100% | 50% | 50% | 0% | 0% | 0% | 100% |
| Hold hands | 100% | 100% | 0% | 0% | 0% | 0% | 100% |
| Hold object | 100% | 0% | 100% | 0% |  |  |  |
| Inspect | 100% | 0% | 100% | 0% | 0% | 0% | 100% |
| Jump | 100% | 63% | 0% | 38% | 0% | 0% | 100% |
| Kick | 100% | 100% | 0% | 0% | 0% | 0% | 100% |
| Kiss | 100% | 20% | 20% | 60% | 0% | 0% | 100% |
| Knock | 100% | 50% | 50% | 0% | 0% | 0% | 100% |
| Limp extend | 100% | 25% | 25% | 50% | 0% | 0% | 100% |
| Linear sweep | 100% | 100% | 0% | 0% | 0% | 0% | 100% |
| Locomote tandem | 100% | 75% | 0% | 25% | 0% | 0% | 100% |
| Lower head | 100% | 0% | 0% | 100% | 0% | 50% | 50% |
| Lunge | 100% | 20% | 40% | 40% | 0% | 0% | 100% |
| Nod | 100% | 100% | 0% | 0% |  |  |  |
| Offer hand | 100% | 0% | 0% | 100% | 0% | 0% | 100% |
| Poke | 100% | 0% | 0% | 100% | 0% | 0% | 100% |
| Pound | 100% | 100% | 0% | 0% | 0% | 0% | 100% |
| Present genitals | 100% | 3% | 29% | 68% | 0% | 0% | 100% |
| Present leg | 100% | 0% | 0% | 100% | 0% | 43% | 57% |
| Present mount | 100% | 8% | 8% | 85% | 0% | 100% | 0% |
| Present rump | 100% | 53% | 12% | 35% | 0% | 29% | 71% |
| Present torso | 100% | 0% | 2% | 98% | 0% | 28% | 72% |
| Pull another | 100% | 20% | 0% | 80% | 0% | 0% | 100% |
| Push by hand | 100% | 13% | 13% | 75% | 0% | 0% | 100% |
| Push by rump | 100% | 100% | 0% | 0% | 0% | 0% | 100% |
| Retrieve | 100% | 0% | 0% | 100% | 0% | 0% | 100% |
| Rock | 100% | 50% | 25% | 25% | 0% | 0% | 100% |
| Roll over | 100% | 0% | 0% | 100% | 0% | 0% | 100% |
| Rub | 100% | 50% | 0% | 50% | 0% | 50% | 50% |
| Run stiff | 100% | 78% | 4% | 18% | 0% | 8% | 92% |
| Shake limb | 100% | 0% | 0% | 100% | 0% | 0% | 100% |
| Shake mobile | 100% | 92% | 4% | 4% | 0% | 6% | 94% |
| Shake stationary | 100% | 25% | 21% | 54% | 0% | 6% | 94% |
| Shuffle | 100% | 100% | 0% | 0% | 0% | 0% | 100% |
| Slap another | 100% | 100% | 0% | 0% | 0% | 0% | 100% |
| Slap object | 100% | 50% | 0% | 50% | 100% | 0% | 0% |
| Slap self | 100% | 100% | 0% | 0% | 0% | 0% | 100% |
| Slide | 100% | 33% | 33% | 33% | 0% | 0% | 100% |
| Smack lip | 100% | 3% | 2% | 95% | 0% | 9% | 91% |
| Sniff | 100% | 67% | 0% | 33% | 0% | 0% | 100% |
| Stamp quadrupedal | 100% | 75% | 5% | 20% | 0% | 0% | 100% |
| Stamp sitting | 100% | 22% | 22% | 56% | 0% | 0% | 100% |
| Stand tandem | 100% | 75% | 0% | 25% | 0% | 0% | 100% |
| Stationary stiff | 100% | 44% | 19% | 38% | 0% | 25% | 75% |
| Stiff extend | 100% | 75% | 0% | 25% | 0% | 0% | 100% |
| Stretched extend | 100% | 67% | 33% | 0% | 0% | 0% | 100% |
| Stroke by mouth | 100% | 50% | 50% | 0% | 0% | 0% | 100% |
| Stroke short | 100% | 0% | 100% | 0% | 0% | 0% | 100% |
| Swagger bipedal | 100% | 86% | 0% | 14% | 17% | 0% | 83% |
| Swagger quadrupedal | 100% | 91% | 0% | 9% | 0% | 29% | 71% |
| Swagger stationary | 100% | 100% | 0% | 0% | 0% | 0% | 100% |
| Sway | 100% | 43% | 29% | 29% | 0% | 14% | 86% |
| Swing | 100% | 73% | 18% | 9% | 0% | 29% | 71% |
| Tap another | 100% | 0% | 33% | 67% | 0% | 0% | 100% |
| Tap object | 100% | 0% | 0% | 100% |  |  |  |
| Thrust genitals | 100% | 100% | 0% | 0% | 0% | 0% | 100% |
| Tickle | 100% | 0% | 0% | 100% | 0% | 0% | 100% |
| Tip head | 100% | 0% | 100% | 0% | 0% | 0% | 100% |
| Touch backhand | 100% | 40% | 30% | 30% | 0% | 0% | 100% |
| Touch innerhand | 100% | 0% | 50% | 50% | 0% | 0% | 100% |
| Touch long | 100% | 33% | 0% | 67% | 0% | 33% | 67% |
| Touch self | 100% | 0% | 100% | 0% | 0% | 0% | 100% |
| Turn back | 100% | 0% | 25% | 75% | 0% | 0% | 100% |
| Turn head | 100% | 0% | 0% | 100% | 0% | 0% | 100% |
| Unilateral swing | 100% | 33% | 33% | 33% | 0% | 0% | 100% |
| Vertical extend | 100% | 15% | 38% | 46% | 0% | 9% | 91% |
| Walk stiff | 100% | 64% | 18% | 18% | 0% | 7% | 93% |
| Wipe | 100% | 0% | 100% | 0% | 0% | 0% | 100% |

If gesture occurred multiple times in a sequence, only one occurrence of each gesture type per sequence was included to compute the gesture frequencies for this table. Bodily orientation during production of first gesture in the sequence or single gesture is given. Blank cells – data missing from this sample of gestures. The bodily orientation of gestures accompanying broadcast panthoot display when gestures were only visual, is the bodily orientation present during the scan sample between the signaller and the most dominant individual in the party. The instances where the gestures were auditory or auditory and visual, the bodily orientation during panthoot is between the signaller and the nearest neighbour during the scan sample. See Supplementary Information 2 for detailed data on observed frequencies.

Supplementary Table 2. Number of entries of single gestures, persistence and rapid sequences per each focal subject (adult to adult gestures only)

| Id of focal individual | Single gesture | Persistence sequence | Rapid sequence |
| --- | --- | --- | --- |
| Bwoba | 54.00 | 0.0 | 7.00 |
| Hawa | 111.00 | 16.00 | 26.00 |
| Kato | 62.00 | 16.00 | 21.00 |
| Kutu | 6.00 | 0.0 | 4.00 |
| Kwera | 4.00 | 4.00 | 2.00 |
| Melissa | 33.00 | 2.00 | 9.00 |
| Musa | 28.00 | 3.00 | 7.00 |
| Nambi | 4.00 | 1.00 | 1.00 |
| Nick | 47.00 | 8.00 | 23.00 |
| Ruhara | 15.00 | 0.00 | 2.00 |
| Squibs | 11.00 | 2.00 | 3.00 |
| Zimba | 10.00 | 1.00 | 2.00 |
