# Supplementary material for: Persistence in gestural communication predicts sociality in wild chimpanzees
Source: Anim Cogn. 2018 Oct 19;22(5):605–18. doi: 10.1007/s10071-018-1219-6 (PMC6689904; doi:10.1007/s10071-018-1219-6)
Supplement: Supplementary file 2 — Supplementary material 2 (DOCX 17 KB) [file 10071_2018_1219_MOESM2_ESM.docx]

**Persistence in gestural communication predicts complex sociality in wild chimpanzee**

**Supplementary Information 2**

**Animal Cognition**

Anna Ilona Roberts, Sam George Bradley Roberts

Supplementary Table 1. Intentionality of gestures by gesture type expressed as frequency of events

| **Gesture type** | **Category of signaller’s and recipient’s bodily orientation during production of the gesture** | | | | | | **Sequence type** | | | |
| --- | --- | --- | --- | --- | --- | --- | --- | --- | --- | --- |
|  | **Recipient facing signaller with side of body, out of signaller’s view** | **Recipient facing signaller with the back, out of signaller’s view** | **Recipient facing signaller with chest, out of signaller’s view** | **Recipient facing signaller with side of body, in signaller’s view** | **Recipient facing signaller with the back, in signaller’s view** | **Recipient facing signaller with chest, in signaller’s view** | | **Rapid sequence** | **Persistence sequence** | **Single gesture** |
| Arm beckon | 0 | 0 | 0 | 0 | 0 | 1 | | 0 | 1 | 0 |
| Arm flap | 0 | 0 | 1 | 0 | 0 | 4 | | 5 | 0 | 5 |
| Arm raise | 0 | 0 | 0 | 0 | 1 | 1 | | 0 | 0 | 2 |
| Beat | 0 | 0 | 0 | 0 | 1 | 1 | | 2 | 0 | 0 |
| Bite | 0 | 0 | 0 | 1 | 0 | 1 | | 2 | 0 | 0 |
| Bob | 0 | 0 | 0 | 2 | 0 | 7 | | 4 | 4 | 3 |
| Bounce | 0 | 0 | 1 | 1 | 2 | 8 | | 3 | 6 | 7 |
| Bow | 0 | 0 | 1 | 1 | 1 | 7 | | 6 | 4 | 3 |
| Break | 0 | 0 | 0 | 0 | 0 | 1 | | 1 | 0 | 0 |
| Clip by hand | 0 | 0 | 0 | 1 | 1 | 1 | | 1 | 2 | 3 |
| Clip by mouth | 0 | 0 | 0 | 1 | 1 | 3 | | 0 | 3 | 3 |
| Crouch | 0 | 0 | 0 | 2 | 0 | 7 | | 4 | 6 | 2 |
| Crouch run | 0 | 0 | 3 | 1 | 3 | 20 | | 26 | 6 | 13 |
| Crouch walk | 0 | 0 | 4 | 4 | 1 | 20 | | 19 | 8 | 12 |
| Dangle | 0 | 1 | 1 | 1 | 0 | 3 | | 6 | 1 | 1 |
| Drag object | 0 | 0 | 0 | 0 | 0 | 0 | | 1 | 0 | 0 |
| Drag self | 0 | 0 | 0 | 0 | 0 | 1 | | 0 | 1 | 0 |
| Drum | 2 | 0 | 1 | 2 | 5 | 5 | | 17 | 1 | 1 |
| Embrace | 0 | 0 | 3 | 3 | 1 | 6 | | 10 | 0 | 3 |
| Forceful extend | 0 | 0 | 0 | 0 | 0 | 2 | | 0 | 1 | 1 |
| Grab | 0 | 0 | 0 | 0 | 1 | 1 | | 1 | 0 | 1 |
| Hand bend | 0 | 0 | 0 | 1 | 0 | 5 | | 2 | 3 | 3 |
| Hit object | 0 | 0 | 0 | 0 | 0 | 1 | | 1 | 1 | 0 |
| Hold hands | 0 | 0 | 0 | 0 | 0 | 1 | | 1 | 0 | 0 |
| Hold object | 0 | 0 | 0 | 0 | 0 | 0 | | 0 | 1 | 0 |
| Inspect | 0 | 0 | 0 | 0 | 1 | 0 | | 0 | 1 | 0 |
| Jump | 0 | 0 | 0 | 0 | 1 | 3 | | 5 | 0 | 3 |
| Kick | 0 | 0 | 0 | 0 | 0 | 1 | | 1 | 0 | 0 |
| Kiss | 0 | 0 | 0 | 0 | 0 | 5 | | 1 | 1 | 3 |
| Knock | 0 | 0 | 0 | 0 | 1 | 1 | | 1 | 1 | 0 |
| Limp extend | 0 | 0 | 0 | 0 | 0 | 8 | | 2 | 2 | 4 |
| Linear sweep | 0 | 0 | 0 | 1 | 0 | 1 | | 3 | 0 | 0 |
| Locomote tandem | 0 | 0 | 0 | 1 | 0 | 3 | | 3 | 0 | 1 |
| Lower head | 0 | 0 | 2 | 0 | 0 | 2 | | 0 | 0 | 4 |
| Lunge | 0 | 0 | 0 | 0 | 0 | 2 | | 1 | 2 | 2 |
| Nod | 0 | 0 | 0 | 0 | 0 | 0 | | 1 | 0 | 0 |
| Offer hand | 0 | 0 | 0 | 0 | 0 | 3 | | 0 | 0 | 3 |
| Poke | 0 | 0 | 0 | 0 | 1 | 1 | | 0 | 0 | 2 |
| Pound | 0 | 0 | 0 | 1 | 0 | 1 | | 2 | 0 | 0 |
| Present genitals | 0 | 0 | 0 | 6 | 3 | 16 | | 1 | 11 | 26 |
| Present leg | 0 | 0 | 3 | 0 | 0 | 4 | | 0 | 0 | 7 |
| Present mount | 0 | 0 | 11 | 0 | 0 | 0 | | 1 | 1 | 11 |
| Present rump | 0 | 0 | 4 | 0 | 0 | 10 | | 9 | 2 | 6 |
| Present torso | 1 | 0 | 11 | 1 | 2 | 28 | | 0 | 1 | 43 |
| Pull another | 0 | 0 | 0 | 0 | 0 | 5 | | 1 | 0 | 4 |
| Push by hand | 0 | 0 | 0 | 0 | 0 | 8 | | 1 | 1 | 6 |
| Push by rump | 0 | 0 | 0 | 0 | 0 | 3 | | 3 | 0 | 0 |
| Retrieve | 0 | 0 | 0 | 0 | 0 | 1 | | 0 | 0 | 1 |
| Rock | 0 | 0 | 0 | 0 | 0 | 2 | | 2 | 1 | 1 |
| Roll over | 0 | 0 | 0 | 0 | 0 | 1 | | 0 | 0 | 1 |
| Rub | 0 | 0 | 1 | 0 | 1 | 0 | | 1 | 0 | 1 |
| Run stiff | 1 | 0 | 2 | 5 | 9 | 19 | | 35 | 2 | 8 |
| Shake limb | 0 | 0 | 0 | 0 | 0 | 1 | | 0 | 0 | 1 |
| Shake mobile | 0 | 0 | 1 | 3 | 2 | 12 | | 23 | 1 | 1 |
| Shake stationary | 0 | 0 | 2 | 7 | 4 | 18 | | 13 | 11 | 28 |
| Shuffle | 0 | 0 | 0 | 1 | 0 | 1 | | 2 | 0 | 0 |
| Slap another | 0 | 0 | 0 | 0 | 0 | 1 | | 1 | 0 | 0 |
| Slap object | 0 | 1 | 0 | 0 | 0 | 0 | | 1 | 0 | 1 |
| Slap self | 0 | 0 | 0 | 0 | 1 | 0 | | 1 | 0 | 0 |
| Slide | 0 | 0 | 0 | 0 | 0 | 3 | | 1 | 1 | 1 |
| Smack lip | 0 | 0 | 12 | 18 | 44 | 65 | | 4 | 3 | 133 |
| Sniff | 0 | 0 | 0 | 0 | 2 | 1 | | 2 | 0 | 1 |
| Stamp quadrupedal | 0 | 0 | 0 | 2 | 4 | 7 | | 15 | 1 | 4 |
| Stamp sitting | 0 | 0 | 0 | 0 | 1 | 7 | | 2 | 2 | 5 |
| Stand tandem | 0 | 0 | 0 | 0 | 0 | 4 | | 3 | 0 | 1 |
| Stationary stiff | 0 | 0 | 3 | 0 | 1 | 8 | | 7 | 3 | 6 |
| Stiff extend | 0 | 0 | 0 | 0 | 0 | 2 | | 3 | 0 | 1 |
| Stretched extend | 0 | 0 | 0 | 1 | 1 | 1 | | 2 | 1 | 0 |
| Stroke by mouth | 0 | 0 | 0 | 0 | 1 | 1 | | 1 | 1 | 0 |
| Stroke short | 0 | 0 | 0 | 0 | 0 | 1 | | 0 | 1 | 0 |
| Swagger bipedal | 0 | 1 | 0 | 0 | 0 | 5 | | 6 | 0 | 1 |
| Swagger quadrupedal | 1 | 0 | 1 | 1 | 2 | 2 | | 10 | 0 | 1 |
| Swagger stationary | 0 | 0 | 0 | 1 | 0 | 0 | | 1 | 0 | 0 |
| Sway | 0 | 0 | 1 | 1 | 2 | 3 | | 3 | 2 | 2 |
| Swing | 0 | 0 | 2 | 1 | 2 | 2 | | 8 | 2 | 1 |
| Tap another | 0 | 0 | 0 | 0 | 0 | 3 | | 0 | 1 | 2 |
| Tap object | 0 | 0 | 0 | 0 | 0 | 0 | | 0 | 0 | 1 |
| Thrust genitals | 0 | 0 | 0 | 0 | 0 | 1 | | 1 | 0 | 0 |
| Tickle | 0 | 0 | 0 | 8 | 1 | 0 | | 0 | 0 | 9 |
| Tip head | 0 | 0 | 0 | 0 | 0 | 1 | | 0 | 1 | 0 |
| Touch backhand | 0 | 0 | 0 | 2 | 1 | 6 | | 4 | 3 | 3 |
| Touch innerhand | 0 | 0 | 0 | 0 | 0 | 1 | | 0 | 1 | 1 |
| Touch long | 0 | 0 | 1 | 0 | 1 | 1 | | 1 | 0 | 2 |
| Touch self | 0 | 0 | 0 | 1 | 0 | 0 | | 0 | 1 | 0 |
| Turn back | 0 | 0 | 0 | 0 | 0 | 2 | | 0 | 1 | 3 |
| Turn head | 0 | 0 | 0 | 0 | 0 | 1 | | 0 | 0 | 1 |
| Unilateral swing | 0 | 0 | 0 | 1 | 1 | 2 | | 2 | 2 | 2 |
| Vertical extend | 0 | 0 | 1 | 2 | 1 | 7 | | 2 | 5 | 6 |
| Walk stiff | 0 | 0 | 1 | 3 | 3 | 7 | | 14 | 4 | 4 |
| Wipe | 0 | 0 | 0 | 1 | 0 | 0 | | 0 | 1 | 0 |
| **TOTAL** | **5** | **3** | **74** | **90** | **112** | **412** | | **319** | **124** | **422** |

If gesture occurred multiple times in a sequence, only one occurrence of each gesture type per sequence was included to compute the gesture frequencies for this table. Bodily orientation during production of first gesture in the sequence or single gesture is given. Differences in total number of events between bodily orientation and sequence types stem from missing data. The bodily orientation of gestures accompanying broadcast panthoot display when gestures were only visual, is the bodily orientation present during the scan sample between the signaller and the most dominant individual in the party. The instances where the gestures were auditory or auditory and visual, the bodily orientation during panthoot is between the signaller and the nearest neighbour during the scan sample.
